# Statistical software applications used in health services research: analysis of published studies in the U.S

**DOI:** 10.1186/1472-6963-11-252

**Published:** 2011-10-06

**Authors:** Allard E Dembe, Jamie S Partridge, Laurel C Geist

**Affiliations:** 1 The Ohio State University College of Public Health, 202 Cunz Hall, 1841 Neil Avenue, Columbus, Ohio 43210, USA; 2 Abbott Vascular, 3200 Lakeside Drive, Santa Clara, CA 95054, USA; 3 The Ohio State University College of Public Health, The Center for Health Outcomes, Policy & Evaluation Studies, 5049 Smith Laboratories, 174 West 18th Avenue, Columbus, Ohio 43210, USA

**Keywords:** Statistical software, data analysis, SAS, Stata

## Abstract

**Background:**

This study aims to identify the statistical software applications most commonly employed for data analysis in health services research (HSR) studies in the U.S. The study also examines the extent to which information describing the specific analytical software utilized is provided in published articles reporting on HSR studies.

**Methods:**

Data were extracted from a sample of 1,139 articles (including 877 original research articles) published between 2007 and 2009 in three U.S. HSR journals, that were considered to be representative of the field based upon a set of selection criteria. Descriptive analyses were conducted to categorize patterns in statistical software usage in those articles. The data were stratified by calendar year to detect trends in software use over time.

**Results:**

Only 61.0% of original research articles in prominent U.S. HSR journals identified the particular type of statistical software application used for data analysis. Stata and SAS were overwhelmingly the most commonly used software applications employed (in 46.0% and 42.6% of articles respectively). However, SAS use grew considerably during the study period compared to other applications. Stratification of the data revealed that the type of statistical software used varied considerably by whether authors were from the U.S. or from other countries.

**Conclusions:**

The findings highlight a need for HSR investigators to identify more consistently the specific analytical software used in their studies. Knowing that information can be important, because different software packages might produce varying results, owing to differences in the software's underlying estimation methods.

## Background

Health services research (HSR) is a highly interdisciplinary field that employs a variety of quantitative and qualitative methods. While many statistical software applications are available to health services researchers, there is no accepted norm in the profession regarding which software product to use for HSR studies. To the best of our knowledge, there is no publicly available software that has been designed specifically for use in HSR studies (although some applications, such as MedCalc, are designed primarily for biomedical analyses). Rather, researchers are free to choose whatever software program is deemed to be most appropriate for use in a particular study, given the study's analytical needs. Additionally, the training and experience of analysts performing the calculations often affects which software is chosen.

This study aims to determine which software packages are most commonly used for HSR studies, based on a review of published articles from U.S. HSR journals. We originally undertook this study to help doctoral students at our university become better informed about some of the major statistical software products that are available, particularly to research trainees in the U.S.

Many leading statistical software vendors, such as SAS Institute, Inc. (Cary, North Carolina), StataCorp LP (College Station, Texas), and SPSS Inc. (a subsidiary of IBM, based in Chicago, Illinois), market a suite of general and specialized statistical software computer programs, branded under such names as SAS, Stata, and SPSS. Spreadsheet applications such as Microsoft Excel (Microsoft Corporation, Redmond, Washington) are also commonly used for statistical analysis. There are also dozens of other statistical software products used for HSR purposes. For example, SUDAAN (RTI International, Research Triangle Park, North Carolina) is a well known software product used for the analysis of complex survey, clustered or other correlated data. A listing of the most widely-used statistical analysis software is provided on several internet sites [1].

Many HSR investigators, particularly junior investigators new to the field, are often uncertain about which software programs to adopt. Attempts have been made by some authorities to evaluate the relative merits and limitations of the various software programs [2-5]. However, the choice of a particular software package for a particular HSR study generally will be dependent on the study's specific computational needs, the investigators' skills and experience, and their judgment about the suitability of a particular software application for a specific analysis.

This study provides researchers with information about the specific statistical software packages that are most frequently used in the HSR field. Three-year trends (2007-2009) in use of statistical software by health services researchers are also presented. Additionally, we document the extent to which published articles contain sufficient information about the type of statistical software used in HSR studies. This study does not attempt to evaluate the relative merits of particular software products for various analytical purposes.

## Methods

Because our focus was on acquainting research trainees in the United States with available statistical software, we selected articles only from U.S. journals. We employed a semi-structured review process to select the journals for the study. Our principal criterion was that the journal be squarely focused on health services research, emphasizing original research articles in that field. However, "health services research" is a broad term that encompasses a variety of research issues in the general health care domain. For that reason, we decided to exclude journals that only focus on a segment of HSR (e.g., *Journal of Pharmaceutical Health Services Research*, *Journal of Health Economics*, *Journal of Behavioral Health Services and Research*), those that focus on management approaches in health care (e.g., *Health Services Management and Administration*), and those that emphasize policy analysis and policy implications of health care research (e.g., *Journal of Health Politics Policy and Law*, *Health Affairs*, and the *Milbank Quarterly*. We also excluded journals with a strong clinical care orientation (e.g., *JAMA *and *NEJM*), public health focus (e.g., *American Journal of Public Health*), and financing/insurance focus (e.g., *Inquiry*, *Medicaid and Medicare Financing Review*).

This resulted in a quite homogeneous set of general HSR journals for our study, of which *Health Services Research*, *Medical Care *and *Medical Care Research and Review *were chosen as representative examples that are well-known to the U.S. HSR community. Arguments could be made for the inclusion of other journals also fitting these criteria. But practical considerations involving the time and money needed to conduct this labor-intensive study (which required careful reading of more than 1,000 articles by our research team) limited our ability to include additional titles.

The investigators obtained copies of every article that was published in *Health Services Research*, *Medical Care*, and *Medical Care Research and Review *during calendar years 2007, 2008, and 2009. In all, there were a total of 1,139 articles published in those three journals combined between January 1, 2007 and December 31, 2009. We assumed that the type of articles most likely to involve the use of statistical software were original research articles. Therefore, we excluded editorials, review articles, commentaries, panel presentations, communications, and meta-analyses. That resulted in the exclusion of 262 articles. Among the remaining 877 original research articles, 342 articles contained no mention of the particular statistical software used. There were multiple reasons why articles failed to identify the specific software utilized: a) some articles mentioned the type(s) of analyses conducted but did not identify the specific software used for those analyses, b) some articles did not describe the type of analyses performed or the specific analytical methods used, and c) some studies employed quantitative or qualitative methods that did not require the use of statistical software. The remaining 535 articles contained at least one mention of a specific statistical software application used for data analysis.

Among the 535 articles, we identified and recorded the specific software application(s) mentioned in each article along with the relevant software version number and release date. However, the exact software procedure used by the researchers was rarely provided and thus we were unable to collect substantial data in that area. Each article was read by one of the study investigators (LG) to obtain and input the data, and a second investigator (JP) re-read the articles and checked the recorded data to verify the accuracy of the entries. We stratified results by journal and by calendar year of publication.

Because the focus of this study was on HSR software usage in the U.S., we did not originally intend to include articles from foreign (non-U.S.) researchers. However, when we began collecting data from each article, we observed that there were large variations in software usage patterns depending on whether the article's authors were foreign or resident to the U.S. This was an unexpected finding and one we thought might have general interest and possibly suggests (by us or others) a more extensive analysis of international trends in statistical software usage for the future. Therefore, we conducted a simple stratification of the data to determine differential results based on whether or not the authors of the article were from the U.S. or from other countries. For that subanalysis, an article with U.S. authorship was defined as one having at least 50% of the co-authors identified as being affiliated with an institution in the United States. All calculations were performed using Excel 2003 spreadsheet software.

## Results

There were 535 articles in which the use of specific statistical software applications was mentioned. Some studies involved the use of more than one type of statistical software application. Overall, among the 535 articles, 637 different instances of statistical software use were mentioned. As summarized in Tables 1 and 2, during the 2007-2009 time period, Stata was mentioned as having been used in 46.0% of all included articles, SAS was used in 42.6% of the articles, SUDAAN was used in 6.2% of the articles, SPSS was used in 5.8% of articles, and a variety of other software applications were mentioned in 18.5% of articles. The latter group represented 34 distinct software applications including MPLUS, MLwiN, R, and HLM.

**Table 1:** Mention of Statistical Software in HSR Articles, 2007-2009, by Year

	2007	2008	2009	2007-2009
Total articles	393	374	372	1139
Excluded articles	111	66	85	262
Included articles	282	308	287	877
Included articles not mentioning software	110	120	112	342
Included articles mentioning software	172	188	175	535
% of all included articles that mentioned software	61.0	61.0	61.0	61.0
Number of software mentions	212	224	201	637
Average number of software mentions per included article	1.2	1.2	1.1	1.2
% of articles in which Stata was used*	48.3	42.6	47.4	46.0
% of articles in which SAS was used*	37.2	43.1	47.4	42.6
% of articles in which SUDAAN was used*	10.5	5.3	2.9	6.2
% of articles in which SPSS was used*	4.7	8.5	4.0	5.8
% of articles in which other statistical software was used*	22.7	19.7	13.7	18.5

* Note: percentages add up to more than 100% because some articles mentioned the use of more than one statistical software application.

**Table 2:** Statistical Software Mentioned in HSR Articles, 2007-2009, by Authorship

	**U.S. Authorship**		Non-U.S. Authorship		**Total**	
	**Number**	**Percent**	**Number**	**Percent**	**Number**	**Percent**
Total included articles	791	100.0	86	100.0	877	100.0
Included articles mentioning software (% of articles)	481	60.8	54	62.8	535	100.0
Included articles mentioning software (% distribution)	481	89.9	54	10.1	535	100.0
Total number of software mentions (% distribution)	574	90.1	63	9.9	637	100.0
Average number of software mentions per article	1.2	--	1.2	--	1.2	--
Articles in which Stata was used (% of articles)*	238	49.5	8	14.8	246	46.0
Articles in which SAS was used (% of articles)*	202	42.0	26	48.1	228	42.6
Articles in which SUDAAN was used (% of articles)*	33	6.9	0	0.0	33	6.2
Articles in which SPSS was used (% of articles)*	17	3.5	14	25.9	31	5.8
Articles in which other software was used (% of articles)*	84	17.5	15	27.8	99	18.5

* Note: percentages add up to more than 100% because some articles mentioned the use of more than one statistical software application.

The proportion of articles in which SAS was used increased steadily from 37.2% in 2007 to 43.1% in 2008 and to 47.4% in 2009. During the same period, the proportion of articles in which use of Stata or SPSS was mentioned remained about the same. The use of SUDAAN fell during this period from being mentioned in 10.5% of articles in 2007 to 2.9% in 2009.

Of the 637 instances of software use, the vast majority (90.1%) appeared in U.S.-authored articles and only 9.9% were in articles authored by researchers from outside the U.S., reflecting the predominantly U.S. orientation of these journals. Although the proportion of non-U.S. authors was relatively small, we observed that the use of particular software products varied considerably depending on whether the authors were from the U.S. or from other countries. For example, Stata was used in 49.5% of articles with U.S. authorship but only 14.8% of articles authored by non-U.S. researchers. Likewise, SUDAAN was used in 6.9% of U.S.-authored articles but in none of the articles authored by researchers from outside the U.S. By contrast, SPSS was used in a much larger proportion of articles with non-U.S. authorship (25.9%) than with U.S. authorship (3.5%). Use of SAS was somewhat more common in non-U.S. authored articles (48.1%) than in articles by authors from the U.S. (42.0%).

## Discussion

There is little information available about what kind of statistical software is used in the HSR field or, for that matter, in other academic disciplines. A variety of articles have been published comparing the features of various statistical software applications and identifying their relative benefits for use in research studies. But virtually no information is publically available about which types of statistical software are most commonly used for data analysis purposes. A few investigators have attempted to offer some insight on this question. For example, a survey conducted by Scotch et al. (2006) found that SPSS was the most popular software used for community health assessment data analysis, followed by SAS [6]. However, that survey was based on a small group of 36 participants. Robert Muenchen (2010) has analyzed data on the number of Google Scholar hits for various statistical software packages from 1995 through 2009 [7]. Muenchen's analysis for 2009 (covering articles from all disciplines) found that Stata was the most commonly cited statistical software application, with about 24,000 Google Scholar hits, followed by SPSS (about 19,000) and SAS (about 17,000).

The present study is the first to provide detailed information on statistical software usage in HSR studies. Our analysis shows that SAS and Stata overwhelmingly are the most commonly used statistical software applications for HSR research in the U.S. Moreover, between 2007 and 2009, the use of SAS increased considerably, while the use of other software applications stayed the same or fell. In particular, use of SUDAAN declined markedly during that period. The decline of SUDAAN and corresponding rise in SAS may have been stimulated by enhancements incorporated into SAS version 9.1.3 (release date: August 31, 2007) and especially version 9.2 (release date: March 14, 2008) that gave users the ability to use balanced repeated replication (BRR) and jackknife methods for variance estimation with complex survey data, in addition to the Taylor series approximation methods that already existed in previous versions of SAS. SAS users thereby gained the ability to apply these advanced methods for estimation of sampling error without needing SUDAAN.

Our simple stratification of the data revealed that use of SPSS was disproportionately greater in non-U.S. authored articles than in U.S.-authored articles (25.9% vs. 3.5%) and use of Stata was disproportionately greater in U.S. authored articles than in non-U.S. authored articles (49.5% vs. 14.8%). Variations based on country of authorship may be the result of various factors, including marketing strategies by the software vendors, researchers' background and training, and the specific types of HSR research conducted in the U.S. compared to other countries. However, since this study was primarily focused on articles from U.S. HSR journals, we were unable to collect sufficient information to determine software usage trends in other countries or to make comprehensive international comparisons. Further studies based on a selection of non-U.S. journals would be needed to draw those conclusions.

Given that we restricted our attention only to original research articles (rather than reviews, meta-analyses, editorials, etc), it was surprising that only 61.0% of articles in U.S. HSR journals identified the specific software used in the data analysis. The proportion of articles mentioning specific analytical software was similar among articles with U.S. authorship (60.8%) and non-U.S. authorship (62.8%). In most cases, there was no way of knowing from the information contained in the articles why the specific type of software used in the data analysis was not identified. We suspect that in some cases the use of statistical software was unnecessary, and in other cases researchers conducted computerized analyses, but without identifying explicitly which particular software application was used. Additionally, some researchers may have written their own computer program to perform an analysis rather than relying on a general-use software application.

It should be noted that journals have varying editorial policies concerning the identification of particular software products. Some journals, such as the *Journal of Preventive Medicine *and the *Journal of the American Dietetic Association*, require authors to specify the name and version number of statistical software utilized. However, some journals specifically instruct authors not to identify the software used for data analysis. For example, instructions for the *Journal of Bone and Joint Surgery *dictate that authors "do not identify any statistical software unless some aspect of the analysis was uniquely dependent on a particular software package." Several authorities have proposed uniform guidelines for reporting of statistical methods and results [8-11]. These guidelines generally advise authors to identify the statistical software and version used, when applicable. Interestingly, none of the three journals used in this study contained specific instructions for authors as to whether or not to identify the specific statistical software employed in their studies.

A primary reason to be concerned about identifying the precise statistical software used in studies is because different software packages can produce varying results, owing to differences in the estimation methods and algorithms used to perform a specific statistical analysis. Indeed, several recent studies have documented numerous inconsistencies in output among commercial software programs based on each program's underlying methodological assumptions [12,13]. McDonald and Vinod (1999), for example, cite a case in which researchers attempting to fit a Cochrane-Orcutt AR(1) correction to a particular data set obtained estimates of ρ (rho) ranging from 0.36 to -0.79 depending on which of four different software packages was used [14,15]. To ensure reliability of analytical results across studies (and reasons for any observed inconsistencies), it would be helpful for investigators to identify not only the general software package and version number, but also the specific software procedures used in the analysis.

## Conclusions

The intention of this brief report is to provide health services researchers with general information about the most common types of statistical software used in HSR, describe recent trends in software usage, identify variations in use of software among researchers in the U.S. and other countries, and bring attention to potential shortcomings in the reporting of information about the specific type of software used. We hope that this information will help researchers during the software selection process and motivate them to provide complete information about specific software employed in HSR studies.

The information should be used with the recognition that our study had certain methodological limitations. For example, some articles contained insufficient information to determine whether or not statistical software was used or which software application was employed. In addition, our analysis did not attempt to qualitatively assess the merits of particular software applications relative to one another or to evaluate their suitability for different analytical uses. Because we used only three HSR journals for the study, results might not be entirely representative of the software usage patterns throughout the entire United States or in specific HSR sub-disciplines, such as health economics. Nevertheless, this study represents perhaps the nation's first attempt to systematically identify the most commonly used statistical software in HSR and provides unique baseline data with which to potentially inform similar attempts to understand software usage practices in other fields.

## Competing interests

The authors have no financial or other competing interests relating to the conduct of this study or its publication.

## Authors' contributions

AD was responsible for the overall design and conduct of this study and was the principal manuscript author. JP oversaw all data collection, was primarily responsible for the data analysis, and participated in the review and authoring of the manuscript. LG collected data, input the data into an analytic data base, and helped to review the manuscript. All authors read and approved the final manuscript.

## Authors' information

AD is Chair of the Division of Health Services Management and Policy at the Ohio State University. In his experience supervising doctoral students and other health services researchers, he has frequently fielded inquiries from investigators about which statistical software packages are typically used, or ought to be used, in the health services research field. JP is an applied economist who, at the time of the study, was a research specialist at the Ohio State University Center for Health Outcomes, Policy and Evaluation Studies (Center for HOPES). LG graduated with a bachelors degree in health sciences from the Ohio State University and currently is a research assistant at the OSU Center for HOPES.

## Pre-publication history

The pre-publication history for this paper can be accessed here:

http://www.biomedcentral.com/1472-6963/11/252/prepub
